# Two Health or Not Two Health? That Is the Question

**DOI:** 10.1128/mBio.00550-19

**Published:** 2019-04-09

**Authors:** William P. Hanage

**Affiliations:** a Center for Communicable Disease Dynamics, Department of Epidemiology, Harvard T. H. Chan School of Public Health, Harvard University, Boston, Massachusetts, USA

**Keywords:** *Escherichia coli*, antibiotic resistance, genomics, molecular epidemiology, One Health

## Abstract

How much drug-resistant infections in livestock contribute to disease in humans is controversial. While zoonoses are a prominent cause of emerging infections, and the profligate use of antibiotics as growth promoters is expected to lead to the spread of resistance, this resistance could remain concentrated in animal pathogens and only rarely spill over into humans. A recent paper compares genomes of Escherichia coli isolates from human bloodstream infections in England, focused on the Cambridge area, with isolates collected from farms and the food chain in the east of the country, seeking evidence of transmission (C. Ludden, K. E. Raven, D. Jamrozy, T. Gouliouris, et al., mBio 10:e02693-18, 2019, https://doi.org/10.1128/mBio.02693-18). While the human and livestock populations were clearly distinct, with very limited evidence for transmission of E. coli or resistance elements to humans, the results also illustrate our limited ability to infer historical transmission events from even the best samples. The implications for the One Health framework, aiming to unify human and veterinary medicine, are discussed.

## Abstract (precis)

How much drug-resistant infections in livestock contribute to disease in humans is controversial. While zoonoses are a prominent cause of emerging infections, and the profligate use of antibiotics as growth promoters is expected to lead to the spread of resistance, this resistance could remain concentrated in animal pathogens and only rarely spill over into humans.

## COMMENTARY

Human life and health are embedded within our ecosystems and methods of food production, which for more than 10 years has been reflected by the One Health Initiative (1). This influential perspective has the stated goal of unifying human and veterinary medicine, emphasizing the importance of zoonoses as a source of emerging human infections and the many networks and interactions between us and animals that contribute to the health we do, or do not, enjoy. No human is an island, nor is any pig, chicken, or cow, to name a few of the animals we have domesticated on industrial scales to feed our exploding population. In a recent article in *mBio*, Ludden et al. examined the potential for domesticated food animals to act as a source of human disease, specifically Escherichia coli bloodstream infections (2).

The One Health framework has been especially compelling when dealing with agriculture because of growing concern about antibiotic resistance. Drug-resistant infections in livestock can lead to drug-resistant infections in humans in three ways: (i) direct acquisition of resistant disease from a livestock source; (ii) transfer of a resistant pathogen to humans, followed by transmission in the human population eventually leading to a case of disease; or (iii) transfer of resistance genes themselves from animal to human pathogens. Livestock have been the recipients of huge amounts of antibiotics, including drugs that are important in human medicine. While the overall dosages can be considered approximately equivalent, we have to account for the much larger combined biomass of the livestock (3). The result is that animals receive far more antibiotics in terms of total mass than humans do, which seems obviously important for the spread of resistant infections in both animals and humans—not least because of the manifold opportunities it affords for new resistance variants to arise. This is not a theoretical concern; the *mcr-1* (for mobilized colistin resistance) gene recently rapidly spread around the world, finding its way through horizontal gene transfer into pathogen and nonpathogen alike. The gene was first detected as dramatically increasing in China, where it was linked to agricultural use of colistin (since banned) (4). While colistin has been an antibiotic no physician would want to use, given better choices (among other things, it is nephrotoxic), it has been indicated in infections highly resistant to other drugs, and the rise of *mcr-1* means it may be taken out of our arsenal sooner than we would like. Even though restrictions on antibiotic use in agriculture have met contested evidence of success, pressure continues to grow for further reductions, especially in the nontherapeutic use of these drugs as growth promoters.

Yet infections, including drug-resistant ones, spread on contact networks. For most humans, as well as most livestock, the overwhelming majority of the contacts we make are with members of the same species, and this has consequences for the spread of disease and resistance. In each case, the relevant selective pressure favoring the spread of resistance, as well as the genes that confer it, is exposure to antibiotic use in that species. Unless people are likely to get their drug-resistant infections directly from the agricultural setting, the way to avoid selecting for resistance is to reduce antibiotic use in humans. The same is true if we want to avoid selecting resistance loci that arose in agriculture, but have then transferred into human pathogens.

Ludden and colleagues (2) examined the overlap between human disease and the bacteria found in agriculture by comparing the genomes of E. coli isolates causing bloodstream infections in English hospitals (predominantly in Cambridge, United Kingdom) with those of isolates from multiple livestock species, sampled from farms in the east of the country, together with a smaller number sampled from the food chain. The question was whether evidence could be found for E. coli isolates from farm or food chain that were closely related to cases of human disease, allowing us to suggest a link between them. The results are both interesting in themselves and because of how they illustrate the challenges to this sort of work.

As we might expect, the great majority of human disease isolates were from known clones or lineages. Molecular epidemiologists call these “clonal complexes,” or “CCs.” In human disease, the most common CCs were 131, 95, and 73, which are the usual suspects in this setting, but all three were completely absent from the livestock sample. In fact, the most common lineages in this sample were associated with either humans or agriculture, but not both, with one exception I discuss in more detail below. This suggests that to a first approximation, the E. coli population responsible for human disease is distinct from that found in agriculture, but this would not rule out the possible transfer of resistance elements between them, as in the case of *mcr-1*. Ludden et al. also find limited evidence for such transfer in this sample, having compared the contigs on which resistance genes were found in the human and nonhuman genomes as a way of comparing their genetic context, looking for overlaps. At first inspection, it looks very much as if this is not so much “One Health” as “>One Health.”

**FIG 1:**
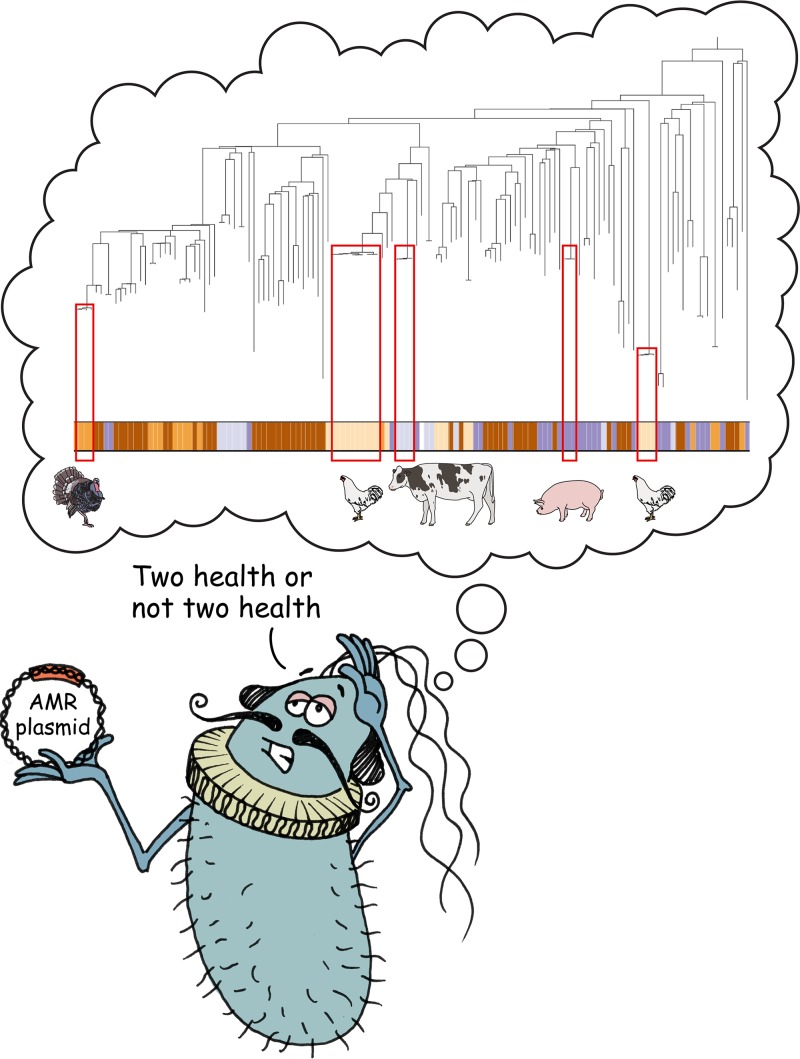
Genomes can be used to produce phylogenies, where the lengths of the branches represent how closely bacteria are related. Almost all the very closely related genomes separated by short branches were isolated from the same host species.

The only major CC to present a more confusing picture is CC10, which combines isolates from multiple sources, but separated by quite long branches on the phylogeny, translating into a distant common ancestor. This is interpreted as ruling out “recent” transmission between humans and the livestock populations sampled in this study. In fact, if we would expect “recent transmission” to look like clusters of very closely related genomes (separated by short branch lengths), then the CC10 results show that these do occur, but they are overwhelmingly isolated from a single host species, frequently on a single farm (Fig. 1). Another CC that contains isolates from mixed sources (CC117) nevertheless shows a deep branch separating closely related subclades associated with different species.

However, we should proceed with caution, because of the varied way the samples were collected. This sort of study design can only hint at the true frequency with which E. coli transmits from humans to animals or vice versa, because it is so unlikely that close links in the transmission chain will be retrieved. The farms involved in this work were relatively closely studied, and so there is plenty of opportunity to detect closely related lineages in them (consider the example of CC10 in the previous paragraph). In contrast, the human sample included patients from a much larger area. While this work presents a truly impressive collection of genomes, it still contains a tiny fraction of the relevant E. coli population. As a result, it is not necessarily surprising that few of the human isolates have close relatives in the data set, neither among E. coli isolates from other humans nor among those from livestock, for the same reason that you’d expect more close relatives in a sample of a hundred people randomly drawn from a small village in the English shires than the same number drawn from the country as a whole. When we have two neighboring tips on the tree, each representing a genome from a different source separated by a long stretch of evolutionary time, it is very hard indeed to say anything about the exact path those two lineages took to the present day, which animals they might have been in, and whether they could have initiated brief outbreaks in unsampled locations—the absence of closely related bacteria in the sample may not be good evidence for their absence in nature. A complementary approach would be to do some deep sampling of both animals and the humans who have contact with them. The finding of closely related genomes in this context would be consistent with spillover, but might say little about the likely future of nascent transmission chains in the alternative host species. However, these issues should not distract from the overall findings of the paper. Transfer of E. coli and their resistance elements from the agricultural setting to human bloodstream infections is not common—at least in England.

Finally, the example of *mcr-1* should remind us not to be sanguine about resistant organisms or genes transferring from agriculture to humans. There are plenty of good reasons to oppose the overuse of antibiotics in agriculture, especially as growth promoters and other nontherapeutic indications, if only to avoid increases in resistant infections afflicting livestock. The findings discussed here may not be replicated with other pathogens, in other environments, or with other disease syndromes (for example, foodborne infections). Farming practices and antibiotic usage are also extremely different in other parts of the world, and this almost certainly impacts the transfer of pathogens and resistance. However, this work is a great example of genome-scale epidemiologic surveillance to examine these questions and to determine whether “tis nobler on the farm to suffer/the slings and arrows of outrageous infections/or to take drugs against a sea of bacteria/and by opposing end them.”
